# Contextual factors influencing advance care planning in home care: process evaluation of the cluster-randomised controlled trial STADPLAN

**DOI:** 10.1186/s12877-022-03026-2

**Published:** 2022-04-20

**Authors:** Katharina Silies, Tilman Huckle, Rieke Schnakenberg, Änne Kirchner, Almuth Berg, Juliane Köberlein-Neu, Gabriele Meyer, Falk Hoffmann, Sascha Köpke

**Affiliations:** 1 Nursing Research Unit, University of Lübeck, Institute for Social Medicine and Epidemiology, Ratzeburger Allee 160, 23562 Lübeck, Germany; 2 Department for Health Services Research, Faculty of Medicine and Health Sciences, Carl von Ossietzky University Oldenburg, Ammerländer Heerstraße 140, 26129 Oldenburg, Germany; 3 Medical Faculty, Institute of Health and Nursing Science, Martin Luther University Halle-Wittenberg, Magdeburger Straße 8, 06112 Halle (Saale), Germany; 4 Center for Health Economics and Health Services Research, Schumpeter School of Business and Economics, University of Wuppertal, Rainer-Gruenter-Straße 21, 42119 Wuppertal, Germany; 5 Institute of Nursing Science, University of Cologne, Faculty of Medicine and University Hospital Cologne, Gleueler Straße 176-178, 50935 Köln, Germany

**Keywords:** Advance care planning, Ambulatory care, Complex intervention, Home care services, Logic model, Mixed methods, Nursing, Process evaluation

## Abstract

**Background:**

The STADPLAN study is a cluster-randomised controlled trial including 27 home care services in Germany. It assesses the effect of an advance care planning (ACP) intervention delivered by trained nurses to older care-dependent patients. Patients received two ACP conversations and an information brochure. Nurses were educated through a two-day programme and topic guides structuring the conversations.

Objectives of the process evaluation were to determine: [1] whether the intervention was implemented as planned, [2] which change mechanisms were observed, [3] whether targeted process outcomes were achieved and [4] in which way contextual factors influenced the implementation process.

**Methods:**

The process evaluation is based on a mixed methods approach following the recommendations of the UK-MRC framework for the development and evaluation of complex interventions. Qualitative and quantitative assessments were developed and analysed guided by a logic model comprising intervention, participants, mechanisms of change and context factors. The results of the main trial will be published elsewhere.

**Results:**

Educational programme and topic guides were mostly implemented as planned and resulted in motivation, knowledge, and perceived competencies to facilitate ACP conversations in nurses. Deviances in the performance of ACP conversations indicated patients’ varied individual needs, but also obstacles like reluctance of patients and caregivers to participate actively and time constraints of nurse facilitators. Patients and caregivers reported increased awareness of ACP, planning and other activities indicating that targeted process outcomes could be achieved. The relevance of multifaceted contextual factors acting as barriers or facilitators for the engagement in ACP interventions on the individual, organisational and macro level was evident.

**Conclusions:**

The process evaluation elicits obstacles and achievements of the ACP intervention. The logic model organised a plethora of mixed methods data into a holistic picture of multifaceted results. Nurses as ACP facilitators in home care can fulfil a crucial initiating role based on a trusting relationship with their patients. To support older care-dependent people’s ACP engagement, access should be simplified.

Furthermore, education for nurse facilitators and sufficient resources for service provision are needed. Independent of monetary reimbursement, healthcare providers must respect patients’ choice for or against any ACP intervention.

**Ethics and trial registration:**

Approved by the Ethics Committees of Martin Luther University Halle-Wittenberg (Ref.-No. 2019–045), Carl von Ossietzky University Oldenburg (Ref.-No. 2019–024), and University of Lübeck (Ref.-No. 19–080).

German Clinical Trials Register: DRKS00016886. Registered retrospectively 04/06/2019, first participant included 29/05/2019.

**Supplementary Information:**

The online version contains supplementary material available at 10.1186/s12877-022-03026-2.

## Background

Advance care planning (ACP) has been defined as “… a process that supports adults at any age or stage of health in understanding and sharing their personal values, life goals, and preferences regarding future medical care. The goal of advance care planning is to help ensure that people receive medical care that is consistent with their values, goals, and preferences during serious and chronic illness” [1]. This goal can be supported by documenting wishes in written documents. Family or persons of trust can be involved in the process [2]. Although ACP has been recommended for every stage of life, it is of specific importance for people facing care dependency or suffering from chronic diseases, where situations in which surrogate decisions may be necessary, are likely to arise. To support the implementation of ACP on a system level, several areas of action need to be targeted, such as policy frameworks, education of healthcare professionals, and reimbursement for ACP services [3]. In Germany, only ACP interventions for care dependent residents in nursing homes and residents in facilities providing integration assistance are covered by the statutory health insurance (Hospice and Palliative Care Act [Hospiz- und Palliativgesetz]). Still, most care dependent people are cared for in their own homes, often supported by family caregivers [4, 5] and/or home care services. These already established contacts with healthcare professionals offer the opportunity to promote ACP at an earlier stage and to a broader group of care dependent people. People living at home are more likely to be capable to fully participate in the ACP process, compared to residents in nursing homes. Nurses in home care build a trusting relationship with their clients and their relatives, which can provide a solid basis for ACP conversations [6]. In addition, the provision of ACP interventions can establish advanced roles for nurses with expanded competencies [7].

The STADPLAN study (Study on advance care planning in care dependent community dwelling older persons) evaluated a complex intervention designed to target this opportunity. It is the first study to provide evidence on the effectiveness of an ACP programme provided by trained nurse facilitators in the home care setting [8]. The intervention’s core components were a training for nurses of home care services (nurse facilitators, NF), a minimum of two ACP conversations based on topic guides with their clients (patients) and an information brochure and workbook on ACP. Patients in the control group received a shorter information leaflet without workbook. Family or surrogates were encouraged to participate in the intervention if patients gave their consent (Table 1, intervention components). The intervention was evaluated in a cluster-randomised controlled trial in 27 German home care services in total, located at three study sites in the northern, western, and eastern part of Germany [8]. The primary outcome was patient activation, measured by the PAM-13 [9, 10], assessing the degree to which individuals take an active role in managing their own health and healthcare and how competent they feel to fulfil that role. The STADPLAN study has been completed and the results of the main analysis will be published elsewhere. This paper reports the results of the comprehensive process evaluation conducted alongside the main trial. By initially analysing and reporting process data before the trial outcomes we follow the recommendations of the UK MRC framework for the development and evaluation of complex interventions in health care [11]. Key to the interpretation of complex interventions is to understand the implementation process in the unique context of the respective study setting and system [12]. Therefore, research questions addressed by the process evaluation were:

1. Was the intervention implemented as planned?
2. Which change mechanisms were observed, were the targeted process outcomes achieved?
3. In which way did contextual factors foster or hinder the implementation?

**Table 1:** STADPLAN intervention components and content

Target group	Intervention component	Content
Nurse facilitators	2-day educational programme	Day 1: ACP basics, aim of the ACP conversations, practical training of the conversation setting and topic guide Day 2: Reflection on experiences and refresher training with case examples
	Topic guide for ACP conversations	Structured guides with main topics, example prompts, and space for noting main results of the conversation
Patients IG	ACP conversation 1	Information on ACP, documentation of patients’ ACP activities to date, introduction to the information brochure and workbook
	ACP conversation 2	Exploration and reflection of patients’ attitudes, preferences and values regarding ACP and health care
	Information brochure and workbook	Information on ACP including a glossary of important ACP concepts and terms, reflexion prompts and contact data of local advisory services
Caregivers IG	Invitation to participate in ACP conversation 2	Listening to the conversation with the patient first and reflecting on the conversations’ results and implications at the end of the conversation
Patients CG	Information leaflet	Short information on ACP and contact data of local advisory services

*ACP* Advance care planning, *CG* Control group, *IG* Intervention group

As integral part of all research questions we considered the role nurses can potentially take over as facilitators in ACP for patients of home care services [13].

## Methods

**Fig. 1:**
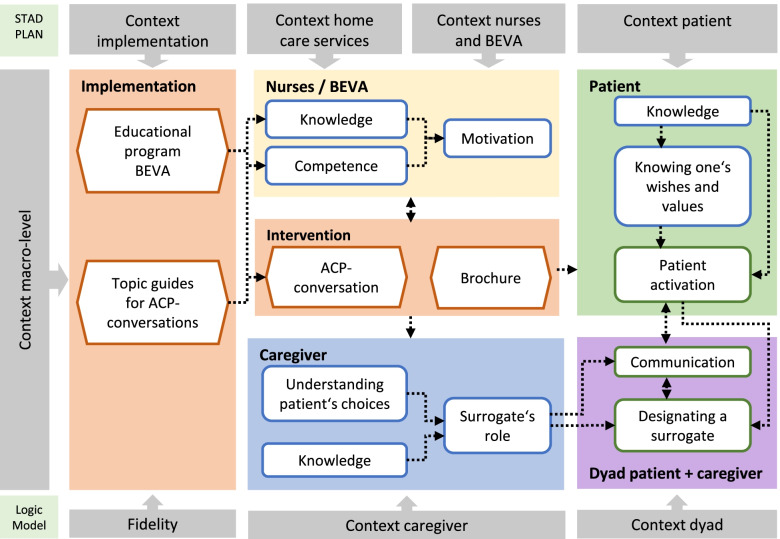
Logic Model. ACP: Advance care planning; BEVA: German acronym for trained nurse facilitator (NF)

Study protocols for the main trial and the process evaluation have been published [8, 13]. The process evaluation is based on a mixed methods approach following the UK MRC framework [11]. Assumptions on how the intervention will induce changes on participants’ and organisations’ levels were depicted in a logic model (Fig. 1). Through the educational programme, nurses are prepared to conduct ACP conversations with patients and their caregivers. Based on these conversations and the information brochure, patients learn about ACP and reflect upon what matters to them in life. Thus, they develop awareness of ACP and are motivated to take action. Caregivers are informed about ACP and develop a better understanding of patients’ choices and of their role as surrogates. Communication on ACP within the dyad of patient and caregiver is facilitated and the designation of a surrogate is supported. A detailed description of the logic model has been published within the study protocol for the process evaluation [13]. Furthermore, we explored contextual factors relevant to the intervention on the individual, the home care services’, and the macro level as indicated in the logic model (Fig. 1), as well as the recruitment process on HCS’ and patients’ level.

### Mixed methods

We used the logic model’s concepts as umbrella structure to relate results across participants and timepoints for the mixed methods analysis. The analysis was based on a pre-designed analysis plan, in which all variables (quantitative as well as qualitative) were ordered according to the logic model’s concepts (implementation, intervention, context and procedural outcomes of participants). On this basis triangulation was performed by comparing quantitative and qualitative results and interpreting them in terms of similarities and differences. Participants, data collection timepoints, methods and assessments are depicted in Table 2.

**Table 2:** Participants, timepoint, method and assessments

Participants (n main trial)	t0	t1	t2	Method	Assessments	Domains of the logic model
*Assessments pre-randomisation*						
Home care Service (HCS)^a^ (*n* = 27)	22			Questionnaire	Basic characteristics of the HCS	Context home care service
Head of HCS^b^ (*n* = 27)	20			Semi structured interview, partly in groups	Experiences with ACP, implementation of ACP in the HCS, motivation and expectation regarding study participation	Context home care service Context nurses Context patients and caregivers
Nurses^b^ (not documented)	22			Semi structured interview, partly in groups	Attitudes and experiences with ACP, motivation and expectation regarding study participation	Context home care service Context nurses Context patients and caregivers
Nurses (not documented)	30			Questionnaire	Formal qualification, work experience	Context nurses
Patients (*n* = 380)	79			Questionnaire	Experiences and expectations regarding ACP and study participation, ACP knowledge, control preferences, satisfaction with HCS	Context patients
*Assessments post-randomisation*						
NF educational programme day 1 (IG)		23		Questionnaire	Evaluation of the educational programme, knowledge on ACP, motivation, and self-efficacy regarding the intervention	Implementation Process outcomes NFs
Educational programme day 1		6		Observation	Duration, alertness of participants and unplanned changes of the programme	Implementation Fidelity
NF educational programme day 2 (IG)		7		5 focus groups, 2 semi structured interviews	Experiences with the intervention and study participation in general	Context HCS Context NFs Implementation Context patients and caregivers Intervention Process outcomes patients and caregivers Process outcomes NFs Process outcomes HCS Macro context
NF educational programme day 2 (IG)		17		Questionnaire	Evaluation of the educational programme, knowledge on ACP	Implementation Process outcomes NFs
Educational programme day 2		5		Observation	Duration, alertness of participants and unplanned changes of the programme	Implementation Fidelity
NF, ACP conversation 1		184		Secondary data extracted from conversations topic guides	Duration Adverse events	Intervention
NF, ACP conversation 2		147		Secondary data extracted from conversations topic guides	Duration Adverse events	Intervention
Patients (IG) (*n* = 210)		41		Questionnaire	Experiences regarding ACP and study participation, ACP knowledge, control preferences, satisfaction with HCS, satisfaction with ACP conversations	Context patients Intervention Process outcomes patients
Patients (CG) (*n* = 170)		42		Questionnaire	Experiences regarding ACP and study participation, ACP knowledge, control preferences, satisfaction with HCS	Context patients
Caregivers (IG) (not documented)	12	11		Semi structured interview	Experiences and expectations regarding ACP and study participation, ACP knowledge, caregiver burden, satisfaction with HCS	Context patients and caregivers Intervention
Head of HCS (CG and IG) (*n* = 27)			22	Semi structured interview, partly in groups	Experiences with ACP, implementation of ACP in the HCS, experiences within the study and further plans regarding ACP	Context HCS Implementation Process outcomes NFs Context patients and caregivers Intervention Process outcomes patients and caregivers Process outcomes HCS Macro context

*ACP* Advance care planning, *NF* Trained nurse ACP facilitator; *CG* Control group, *HCS* Home care service, *IG* Intervention group. T1: six months after t0, t2: twelve months after t0

^a^Basic characteristics at t2 not included into the analysis due to low response (10 of 27)

^b^In some home care services head nurses and staff wanted to be interviewed together (*n* = 7). We excluded these interviews from the analysis because of the methodological difference in the data collection

### Sampling

We followed a combined sampling strategy. On HCS level (basic characteristics, heads of HCS and nurses) we strived to collect a 100% sample. Nurses were eligible as NF if they had at least a 3-year vocational training in nursing (inclusion criterion of main trial). On patients’ level we aimed to ensure all regions and HCS were represented, therefore we recruited patients from every HCS (purposive sampling, aim four patients per HCS). To participate in the main trial, patients had to be at least 60 years old and care-dependent classified by the German statutory health insurance. Participating in the process data collection was an additional effort not every patient was capable of or prepared to do. Therefore, among the patients of one HCS we recruited a convenience sample. Caregivers were recruited from the intervention group only, as interviews solely focused on caregivers’ experiences with the intervention (one caregiver per HCS).

### Data collection

Data were collected at three time-points: t0 (pre-randomisation), t1 (6 months after t0) and t2 (12 months after t0). For basic characteristics of HCS, we asked heads of HCS to fill in data sheets. For patients, we used closed and open-ended questions in face-to-face meetings at t0 during the main data collection and telephone interviews at t1. Caregivers were interviewed by phone. Heads of HCS and staff were interviewed face-to-face or by phone depending on interviewees preferences and schedule (Table 2, participants, methods, and assessments). Interview guides were piloted prior to the main study and implemented without major amendments (Additional file 1, interview guides). A detailed description of the topic guide development is available in the study protocol [13]. Interviewers were trained and experienced researchers employed at the collaborating universities and not involved in any previous professional or personal relationships with participants. All interviews were conducted in German.

In addition to the primary data, the topic guides of the ACP conversations were used as secondary data source to extract the duration of the conversation and adverse events.

### Data processing

All interviews were audiotaped and transcribed verbatim. We employed SPSS (IBM SPSS Statistics for Windows, Version 22.0) and Microsoft Excel (Microsoft 365) for processing quantitative and MAXQDA plus 2020 [14] for processing qualitative data. Data protection was performed in accordance with participating universities’ requirements and approved by universities’ data protection supervisors. Translation of quotes for publication purposes was performed by KS and controlled by SK, who are both native speakers of German with excellent English language skills.

### Data analysis

We analysed quantitative data descriptively (frequencies, percentages, means, range) and used thematic and content analysis to categorise and summarise answers to open-ended questions in patients’ questionnaires. For all interview data we used a qualitative two-step approach including a descriptive and an analytic phase. In the descriptive phase, we built a thematic framework to organise and summarise data separately for each data set (the sum of data collected in one participant group and time point) [15]. A code-system was developed in an inductive-deductive manner: first-level constructs of the coding-systems were defined as the elements of the logic model, whereas second-level constructs were derived from the data and arranged according to first-level constructs (code-systems provided in additional file 1). Data were summarised on interview and code level (‘summary grid’). The analytical phase encompassed clustering results per code and connecting data to the research questions of the process evaluation. We aimed to deal with the large amount of qualitative data in an efficient but appropriately objective manner. Therefore, a primary coder performed all qualitative analyses in the descriptive phase. This researcher developed the code-system, coded and summarised data, keeping close to original wordings. The steps were repeatedly (at least thrice) reviewed by a second researcher (KS and TH acting as primary coder and reviewer and vice versa) and finally discussed with a senior researcher (SK). In the analytical phase we discussed the summarised data in repeated sessions with the extended research group (all authors) to ensure a detailed interpretation of the results in relation to the research questions. All researchers were experienced in qualitative research in process evaluations.

### Trial registration

German Clinical Trials Register: DRKS00016886. Registered retrospectively 04/06/2019, first participant included 29/05/2019.

## Results

In the following section we summarise the results according to the elements of the logic model: a) context of HCS and nurses, b) implementation (education on NF level), c) process outcomes of NFs, d) context of patients, caregivers, and the dyad, e) intervention (ACP conversations and brochure), f) process outcomes on individuals’ levels and on the level of HCS, and g) context macro level (Table 3, summary of results). Initially, we describe the context of the study execution. Quote references indicate participants (nurse facilitators (NF), heads of HCS (Head), caregivers (CG) and patient (P)) and timepoint (t0, t1, t2).

**Table 3:** Summary of results

Positive / facilitating factors	Negative / hindering factors
**Study context**	
• HCS were satisfied with the conduct of the study and the collaboration with the universities • Participating HCS were highly motivated to test a new intervention to improve service for their patients.	• Recruitment difficulties on the level of HCS (resource scarcity) • Recruitment difficulties on the level of patients (main reasons: not interested in the topic, felt sufficiently prepared, topic to burdensome) • The SARS-CoV-19-pandemic interfered with the STADPLAN study regarding the intervention and data collection.
**Context of nurses and home care services**	
• NFs had the qualification and experience required. • NFs had a positive attitude towards ACP and were motivated to perform the intervention. • Conversations on advance directives and power of attorney partly established in HCS or activities planned.	• NFs anticipated obstacles on patient level and had doubts regarding the applicability of ACP in the home care setting. • Organisational barriers (resources and workflow).
**Implementation**	
• Both workshop days were performed as planned. • All NFs were reached at day 1 • NFs were highly satisfied with the workshops.	• Not all NFs present on day 2, due to illness, workload and change of employer.
**Process outcomes NFs**	
• Knowledge, self-perceived competencies, and motivation on NFs level reached.	• NFs described feelings of insecurity and doubts. • NFs anticipated obstacles regarding caregivers’ and patients’ acceptance of the intervention.
**Context of patients, caregivers, and the dyad**	
• Patients and caregivers describe high satisfaction with HCS and trusting relationship. • Open-mindedness of caregivers and patients for ACP.	• NFs observed difficult decision-making processes in families. • High variety of contextual factors on patients’ and caregivers’ level. • Deviating perception of NFs and caregivers regarding caregivers’ engagement in decision-making and ACP.
**Intervention**	
• Intervention mostly implemented as planned. • NFs developed strategies to overcome obstacles. • Patients and caregivers were mostly satisfied. • Information brochure rated as useful by most participants.	• Not all patients reached, not all patients received two conversations • Some conversations too short for in-depth reflection and communication. • Obstacles on level of patients (acceptance and capability to participate), NFs (competencies and personality) and organisations (resources). • Conversations and information brochure too complex for some patients. • Adverse events (three patients stopped participation feeling overburdened by data collection or intervention).
**Process outcomes individual level (patients, caregivers, and dyad)**	
• Patients felt well informed and gained clarity about their wishes. • Patients’ increased awareness and activities like communication, documentation reported by all participant groups. • Caregivers reported deeper understanding and conversations in the dyad, reflection, awareness and plans for activities.	• Patients and caregivers describe that the intervention had no additional benefit or made no changes for themselves and the dyad. • NFs reported caregivers were difficult to integrate in conversations (time constraints, patients refusing to involve them). • NFs reported persistent insecurities and deviances in decision-making in dyads.
**Process outcomes on the level of HCS**	
• Heads of HCS and nursing staff better informed and aware of ACP, motivated to further implement the topic. • Organisational changes like redesigning the assessment of patients’ ACP activities and documents took place. • Plans for further activities regarding ACP services in the organisation were described.	• HCS and nurse staff were severely disappointed to be randomised into control group. • Study participation was too demanding and time consuming.
**Context macro level**	
• HCS are an important access point to ACP. • Interprofessional and trans sectoral collaboration supports ACP and treatment according to patients’ preferences. • The general population develops increasing awareness of the relevance of ACP and palliative care.	• Currently, ACP services are fragmented and access for people with impaired health or care-dependency is too burdensome.

*ACP* Advance care planning, *HCS* Home care service, *NF* Nurse facilitator

### Study context

#### Recruitment of home care services

The recruitment of HCS was documented at each study site. The recruitment aim of 32 clusters with 960 patients was missed by a large margin. Study sites spent massive effort on contacting HCS, prolonged the recruitment phase and widened catchment areas. On average, about 10% of HCS contacted agreed to participate. The main reason for non-participation was lack of resources (qualified nurses and finances), although reimbursement for participation was offered.

#### Recruitment of participants

HCS were asked to document recruitment of participants and reasons for non-participation anonymously. Fifteen HCS documented recruitment contacts with 632 patients of which 403 declined and 229 agreed to participation (recruitment success 36%, comprising more than half of HCS and participants). Reasons for non-participation most often documented were “not interested” (*n* = 92), “already well prepared” (*n* = 90), “too burdensome” (*n* = 39) and “too difficult to understand” (*n* = 12). Some heads of HCS described recruitment as time consuming and explaining the study topic and design as too complicated for patients. Recruitment success varied considerably between clusters (range 5 to 27 patients), regardless of the total size of the HCS.

In summary, the evaluation of the study execution shows that HCS are under considerable strain due to tight financial margins and lack of qualified personnel. This impairs study participation as well as implementation of ACP services in the home care setting. Recruitment difficulties on patients’ level underline that ACP engagement is largely dependent on personal preferences. Still, the average recruitment success shows that a considerable number of patients was interested in a free of charge ACP service.

### Context of nurses and home care services

Twenty-seven HCS were included in the study. One HCS was a public, 12 were non-profit and 14 were for-profit organisations. Twenty-one HCS provided data for the process evaluation at t0. On average these HCS cared for 186 patients (range 17–717) and had an average number of staff of 54 persons (range 17–210). We asked HCS to nominate nurses for participation in the study as trained nurse facilitators if the HCS was randomised to the intervention group. Thirty nurses nominated participated in (group-)interviews at t0. On average they were 43 years (mean, range 23 to 63 years) and had 22 years of work experience (mean, range 7 to 38 years). Eighteen nurses had additional qualifications, for example training in palliative care.

When asked for their motivation to participate in the study, they described a positive attitude towards ACP and underlined its relevance for the home care setting. They aimed to develop their own competencies and knowledge, to enhance awareness of ACP, and to strengthen a trusting relationship with patients and relatives.

*“I wish indeed that things can be specified, that a clear path can be developed. That I can give patients a feeling of safety and inform them on what is possible in this situation. Yes. I hope for training, so I can help patients better and also feel more self-confident.”* (NF_08_t0)

Furthermore, they underlined the necessity to document patients’ status of ACP (wishes and documentation) in the HCS. Yet, they also anticipated obstacles on the organisational level, like lack of time and personnel, and on patients’ level, like unwillingness to discuss the topic or to change their attitude. General doubts related to the usability of advance directives in home care.

*“Me, as a nurse, I have not much to do with advance directives. I just can’t enforce others to use it. Its doctors’ responsibility. But I can think of a situation where an advance directive was documented. But not followed at all.”* (NF_01_t0)

In summary, although obstacles were described, we interpret the contextual factors of the participating home care services and especially the qualification and motivation of the nurses as mostly supportive for the implementation of the intervention.

### Implementation (education of NF)

We performed day 1 of the educational programme seven times in total at three study sites and all 23 assigned NFs of 14 HCS in the intervention group participated. After this workshop day, NF were prepared for and started conducting ACP conversations. On day 2 of the programme 17 NFs participated (loss due to illness, workload or change of employer). Small groups (max 5 participants per workshop day) provided a supportive working atmosphere and constantly alert participants. All workshops were led by at least two trainers from different study sites. In all day 1 workshops, trainers assigned slightly less time to training of conversations as planned, spending more time on the theory and discussion of ACP interventions.

In retrospect, NFs reported to be very satisfied with the overall conduct of the programme and felt motivated and well prepared for own ACP conversations.

*“I think it [workshop day 1] also had a motivating character. A feeling like, “ok, let’s do it”.* (FG_05_t1)

Quantitative results show that NF had high knowledge about ACP and felt competent to explain the topic to patients and to conduct conversations on difficult topics (Table 4, questionnaire-items provided in additional file 1).

**Table 4:** Process outcomes on NFs’ level (quantitative)

Domain		Day 1, *n* = 23	Day 2, *n* = 17
Knowledge (Five Items, number of correct answers, missings counted as wrong)	Mean Median	4.5 5	4.1 4
Competence (perceived self-efficacy) (Eight items, answers from 1 to 6, 1 = feel very capable, 6 feel not at all capable …)	Mean Range	2.0 1–5	1.7 1–4
Motivation (overall positive expectation) (One item, answers from 1 to 6, 1 = feel very optimistic, 6 = feel not at all optimistic)	Mean Range	1.7 1–3	N/A
Overall satisfaction with workshop (One item, answers from 1 to 6, 1 = feel very satisfied, 6 = feel not at all satisfied)	Mean Range	1.3 1–2	1.4 1–3

NFs rated the topic guides as helpful for the training as well as for the conduct of the ACP conversations. Also, they used individual strategies, e.g. starting conversations with patients they felt more comfortable with.

*“Well, in my case, in the first conversation I was really nervous ( … ), but the topic guide helped a lot. Ok, my first conversation was with, like the ideal patient. I chose her on purpose. But then, you get more self-confident with every conversation, I think.”* (FG_04_t1)

In summary we interpret the implementation of the educational programme and topic guides as successful. NFs felt well prepared for the conduct of the intervention regarding their knowledge, competence in leading conversations and motivation. Although they were aware of potential obstacles, they anticipated strategies to deal with them.

### Context of patients, caregivers, and the dyad

We explored contextual factors on patients’ and caregivers’ level related to their experiences and attitude towards ACP, as well as their relationship in the dyad and with the HCS. Sociodemographic characteristics of patient and caregiver participants are displayed in Table 5.

**Table 5:** Patient and caregiver participants (at t0)

Participant	Age (mean)	Women	Care dependency^a^	Living situation
Patient Main trial (*n* = 380)	79.9	66.8%	Low: 73.7% Medium: 23.4% High: 0.3% Unknown: 2.6%	N/A
Patient Process evaluation (*n* = 79)	77.7	64.6%	Low: 74.7% Medium: 24.1% High: 0.0% Unknown: 1.3%	Alone: 69.6% Cohabitating: 30.4%
Caregiver (*n* = 12)	67.9	83.3%	N/A	With patient: 58.3% Near patient (< 2,5 km): 33.3%

^a^Low: German grades 0/1/2; Medium: German grades 3/4; High: German grade 5, as assessed by expert raters of the German statutory health insurance

#### Patients

**Patients** described ACP and autonomy in general as important topics. They were highly satisfied with their HCS, and the reason most often mentioned by patients as motivation for participation was a trusting relationship with the nurses. Asked for their knowledge on ACP, patients had a good understanding of what advance directives are used for. Less well known were prerequisites for surrogate decision making. Some patients described ACP as difficult topic which they procrastinated.

*“I am delaying it, this advance directive, it burdens me. It is negligence. There are many things I don’t manage sometimes. It is also rather negative. I am delaying it.”* (P_57_t0)

Caregivers viewed patients as open-minded, but they also rated illness, increasing frailty and care dependency as barriers for patients’ engagement in ACP. NFs stated a heterogeneous status of patients’ ACP activities. Sometimes patients seem unaware of the relevance of ACP and defer responsibility for decision-making to caregivers. But they also described facilitators like patients’ higher education, experience with death and dying, supportive family and trusting relationship with nurses. NFs, like caregivers, named patients’ hesitancy to talk about dying, a limited access to advisory services, and impaired cognitive abilities as barriers.

#### Caregivers

**Caregivers** felt well informed about ACP and described a variety of information sources, yet also stated a need for deeper understanding. Some felt openminded, others described procrastination or stressful situations like family conflicts hindering communication on ACP.

From NFs’ perspective, caregivers needed information and support to set up ACP documents. They had the impression that caregivers valued present care arrangements and financial aspects more than discussing patients’ ACP. Caregivers’ engagement could also be motivated by seeking justification for decisions they have to make for the patient.

Caregivers did neither voice explicit expectations regarding the ACP intervention nor regarding nurses as ACP facilitators. They assumed that nurses might be suitable facilitators because of their friendliness, inclination to help, ability to explain information and the regular contact in home care. But they also doubted that nurses had enough time resources and were aware of varying levels of nurses’ competencies.

*„Yes, or the information given is not that [complete] by some of them [nurses], it depends on who provides care, it depends on the individual person, some are really competent and excellently trained and well informed and they could probably pass this information on very well. With others it might be better to have a nurse specialist or a nurse manager, who specifically covers this field.”* (CG_05_t0)

#### Dyad of patients and caregivers

In the **dyad of patients and caregivers** we focused on their communication on ACP and decision-making. A longstanding and trusted relationship provides a good basis for ACP, but caregivers also described conversations, which were not deep or detailed enough. NFs and heads of HCS observed lack of communication, from patients’ side because they did not want to be a burden to caregivers, and from caregivers’ side because they refused to think about their loved ones’ death.

Caregivers reported a panorama of decision-making habits in the dyad. Some patients are capable to decide on their own, others prefer caregivers to take over. NFs and heads of HCS observed difficulties in decision-making in dyads and pointed out that relatives were frequently reluctant to take over responsibility.

*„And then we have these patients with caregivers who say ‘I do not want to decide’. ( … ) they have a good relationship within the family and caregivers would in theory be capable to take care of things, but they refuse it and prefer to nominate an external legal guardian.” (*Head_16_t2*)*

In summary, participants’ diverse perspectives illustrate a high variability of patients’ and caregivers’ context factors and multiple aspects possibly facilitating or hindering engagement in ACP. Most participants acknowledged the necessity of ACP and were prepared to get involved. NFs need to account for unique constellations in each patient, caregiver, and dyad which can be challenging in ACP conversations.

### Intervention

The core intervention consisted of a minimum of two guided ACP conversations and an information brochure.

#### ACP conversations

NFs documented 184 first conversations and 147 second conversations. The duration was highly variable (range 10 to 120 min in both conversations). Mean duration of the first conversation was 36 min (SD 19), mean duration of the second conversation was 49 min. In one HCS the second conversation was notably shorter (mean 20 min, SD 11 min, *n* = 21 second conversations documented). NFs absent at day 2 of the workshop had either completed the intervention or handed over to a NF colleague of the same HCS.

NFs described positive aspects of the intervention: they were able to create appropriate surroundings, felt well prepared, and experienced conversations as motivating and valuable. On the other hand, they also found conversations too long and exhausting and felt their motivation decrease over time. They found it partly difficult to include caregivers. For some NFs, as well as for some patients, it was demanding to talk about death and dying. NFs developed strategies to overcome barriers, for example by using workshop material to prepare and to deal with their own uncertainty. Other strategies were taking enough time for conversations, turning off their phone or limiting the number of conversations scheduled per day. From HCS heads’ viewpoint, barriers evolved on all levels: On NFs level, self-management, motivation, and personality were crucial. Some patients were reluctant to engage in conversations or did not have time. The season (December), the overall workload, external quality audits and the SARS-CoV-19-pandemic were additional obstacles that HCS heads described to have interfered with the implementation of the intervention.

Patients described overall satisfaction with ACP conversations. Caregivers outlined how patients felt heard and strengthened in their perception of self-determination.

*„It was indeed, that someone was interested in her ideas, how she imagines things to be, that it is clear and also important that it is about her expectations, that she can decide on her own how things should be in the end.”* (CG_01_t1)

Other caregivers would prefer to talk about ACP in the family or informally, rather than with ACP facilitators in formal conversations.

NFs found patients expressed gratitude and satisfaction with the conversations, but some were hesitant to engage in deep conversations and preferred to talk about current care topics.

*“Yes, they were very grateful. The conversations were very open. It was another point adding to trust, you enhance the trusting relationship with the patients that you already have.”* (FG_05_t1)

*„The second conversations are more intense, and you have to keep the topic guide in mind, to avoid being distracted, left and right, by other problems that are present in current care. ( … ) It is difficult to differentiate [between general nurses’ and NFs’ roles], therefore I think it would be better to have external consulting experts, because it [ACP] gets mixed with current care [problems]. Or patients undoubtedly return to other things repeatedly instead of sticking to the topic [of ACP].”* (FG_01_t1)

#### Information brochure

About half of the patients read the brochure but only a few used it for notes or further conversations. NFs mentioned positive feedback from patients and found it useful for themselves. At the same time, they rated it as less appropriate for patients who do not regularly read longer texts and are less educated.

*“I had participants from two very different parts of the town. And I just have to tie that a little bit to their life story and education. That it was really helpful for those with certain professions. But I also had a group that was more from the working class, they were extremely overwhelmed ( … ), and did not use it. So, for me it was also helpful, but I think it simply requires certain skills to be able to deal with it, which were not available to everyone. ( … ).”* (FG_05_t1)

Caregivers similarly rated the brochure as well designed but also pointed out difficulties of older patients due to impairment and illness to deal with this kind of information.

In summary, we interpret most conversations as implemented as planned, even if there were restrictions in reach, dose, and fidelity. Obstacles occurred on the level of NFs, organisations, patients, and caregivers alike. Many participants expressed their satisfaction with the intervention in detail. Yet, the broad spectrum of reactions raised by the intervention indicates that it was not suitable for every participant and must be tailored to individuals’ needs.

### Process outcomes on individuals’ levels and on the level of HCS

In patients and caregivers, we focused on knowledge of ACP, knowing and understanding patients’ wishes and values, and raising awareness and activities for ACP. In the dyad, we focused on communication, decision-making and designation of a surrogate.

#### Patients

Twenty-two of 32 patients felt they could understand the aims of ACP better after the intervention, although patients of the control group answered questions on ACP knowledge more often correctly. Overall, patients showed moderate knowledge about ACP, which did not vary largely between groups and timepoints (Table 6).

**Table 6:** Process outcomes on patients’ level

Domain		t0 (*n* = 79)	t1 IG (*n* = 41)	T1 CG (*n* = 42)
**Knowledge** (5 Items, number of correct answers, missings counted as wrong)	Mean Median	2.8 3	2.6 3	2.8 3
**Knowing one’s wishes and values** (One item, answers from 1 = strongly agree to 6 = strongly disagree); *n* = 32	Mean Range	N/A	2.2 1–6	N/A
**Overall satisfaction with ACP conversation** (One item, answers from 1 1 = very satisfied, 6 = not at all satisfied); *n* = 31	Mean Range	N/A	1.9 1–4	N/A

*ACP* Advance care planning

Heads of HCS and NFs described a clarifying effect of the intervention and uncertainties of patients and caregivers being remedied. One caregiver described how her husband made a differentiated decision on life-sustaining treatments based on knowledge retrieved in the ACP conversation. Furthermore, the conversation had supported herself to advocate for her husbands’ wishes.

*„It made a difference, this conversation. ( … ) I think I would not have managed that way. ( … ) And my husband, he understood somehow, otherwise he wouldn’t have agreed to it [ventilation]. ( … ) And I am grateful, that she [NF] talked with us about it and my husband agreed to it, otherwise he would not be here today.”* (CG_05_t1)

Twenty-one of 30 patients rated conversations as supportive to gain clarity about their own wishes. Participants’ multiple perspectives show, how patients were activated to engage in ACP. A patient stated:

*“It is important to talk to a trusted person about my wishes. My daughter participated and it was a possibility to talk more deeply and explicitly about it than before.”* (P_03_t1)

Heads of HCS described a variety of changes induced by the intervention on the level of patients regarding awareness, activation, communication, and knowledge. A NF described how the conversations motivated a patient to reflect upon ACP and to talk to her children:

*„Yes, I had one patient, she didn’t have anything, and we went through the brochure and the next time she had written down questions for me which we looked at. ( … ) And now she has a power of attorney and really has jointly met with all her children. She always had this power of attorney, this blank form and repeatedly delayed filling it in and I asked her again and again. But now it worked out.”* (FG_02_t1)

#### Caregivers

Caregivers stated they were already well informed about ACP but gained additional information and a deeper understanding of patients’ wishes. Conversations forced them to deal with the topic and their role as surrogates. They described reflection, raised awareness, and plans for further activities like communicating with the wider family.

#### Dyad of patients and caregivers

A caregiver described how the intervention facilitated communication in the dyad.

*„Well ( … ), that we talked about it at all. Of course, what was new, when she feels unwell, ‘should we call an ambulance or what would you like, would you like to be treated, would you like to have medication’ ( … ). And we have never talked about it that openly before, and certainly not with persons outside the family. Not even with her physician. They never had time for it.”* (CG_01_t1)

From NFs’ perspective, it was possible to stimulate communication in the dyad and to clarify surrogates’ insecurities. Yet, it could be difficult to engage family or caregivers in the ACP conversations. Dyads’ engagement in ACP was strongly shaped by their established manner of communication and their longstanding relationship.

Still, patients and caregivers also described that the intervention had no additional benefit or made no changes for themselves and the dyad.

#### Level of home care services

In some HCS the topic was presented in team meetings and staff engaged in the recruitment of patient participants. Heads of HCS reported they felt more aware of the topic and observed this also in nurses who were not directly participating as NFs. One HCS in the control group started developing an own concept for a healthcare planning service including aspects of ACP. Some heads of HCS reported they had changed documentation protocols to support regular documentation of patients’ ACP activities. Others described plans for future activities like offering further education for nurses, using study materials, and developing ACP services reimbursed by private financing.

In summary, participants described changes in targeted process outcomes on the individual level, which we interpret as indicating that the intended mechanisms of change could take place. Still, individual context factors were key to the impact of the intervention. Additionally, participants described changes the study induced on the level of the organisation. Documentation of surrogate decision-makers and powers of attorney was important for HCS and could be implemented with small effort to further raise awareness of the topic in nurses, patients, and caregivers.

### Context macro level

We focused on two aspects of the macro context, the current situation of ACP in the health care system and the impact of the Covid-19 pandemic. The current situation encompasses the role of HCS and other providers for ACP provision, and the awareness for the topic on societies’ level. NFs and heads of HCS rated the role HCS should play in providing ACP controversially. HCS are an important early access point as older people are increasingly care-dependent and cognitively impaired when admitted to nursing homes. For HCS it is also important to have detailed knowledge of patients’ status of ACP to tailor their service according to patients’ wishes. For nurses, providing ACP offers the possibility to create a deeper relationship with patients and caregivers which was appreciated for both sides.

*„So, the study in general was good for the patients, but also for me. ( … ) you don't talk about such things in nursing, right? ( … ) you know more about the patients afterwards, in the end, after the second conversations. And you don't just talk about these questions, as it says in here [topic guide], you also generalise, you talk about this and that. It gives quite a bit more closeness, I would say. “*(FG_02_t1)

On the other hand, they named scarce time resources and lack of qualified personnel as severe barriers. To be gatekeepers for ACP and refer to ACP providers outside the HCS seemed to be a sound alternative. Yet, currently services are fragmented making access difficult especially for older and care-dependent patients.

*“That was exactly the question, with whom can I do this? ( … ) And of course, that is even a bigger hurdle, if it is separated and you say that one [provider] offers this and the other offers that, then nobody will do it in the end. Because that is just too burdensome. For many [patients], the conversation [in the study] was already burdensome*. (FG_03_t1)

In general, from nurse participants’ viewpoints, ACP is still perceived as a difficult topic, due to the connection with death and dying. At the same time, they observed an increasing acceptance for palliative care and rate ACP as a joint service of palliative care teams, home care services and physicians as promising.

We assumed that the SARS-CoV-19-pandemic might have enhanced participants’ engagement in ACP, as the intervention was still ongoing in the spring of 2020. Yet, NFs and HCS noted varied reactions of patients. For themselves, they observed an increased awareness of the topic, although ACP conversations were hampered by contact restrictions and some interventions could not be completed as planned.

Interpretation: Structures and service portfolios of HCS are varying and providing ACP is not an option for each organisation. Yet, HCS offer an excellent access to patients in need of ACP and could act as important gatekeepers. To provide an accessible and comprehensive ACP service, collaboration of different providers needs to be improved. Participants perceived the awareness of the population in general as increasing which might foster further implementation of ACP.

## Discussion

The process evaluation describes the implementation of the ACP intervention, process outcomes achieved, and the major role of contextual factors.

Educational programme and topic guides were mostly implemented as planned and resulted in motivation, knowledge, and perceived conversational competencies to facilitate ACP conversations in participating nurses. Most ACP conversations with patients were performed as planned. On the other hand, short duration of some conversations and missing second conversations indicate incomplete ACP processes. Deviances may be due to patients’ varied individual needs, but also reveal obstacles like reluctance of patients and caregivers to participate actively, and time constraints of NFs. Patients and caregivers reported increased awareness of ACP, planning, and other activities which show that in principle it was possible to achieve the intended process outcomes. The degree to which these outcomes could be reached remains unclear. Recruitment difficulties on the level of HCS showed how resource scarcity might hamper the implementation of additional services. Recruitment difficulties on patients’ level show that participation of an ACP service may be largely dependent on individual preferences and conditions. The relevance of multifaceted contextual factors for engagement in ACP interventions on the individual, organisational and macro level became evident.

### Individual level

An important facilitator helping patients to engage in ACP in our study was the trusting relationship patients felt towards “their” nurses. Patients and caregivers expect healthcare professionals to initiate ACP [16] and this initiating role has been shown to be strongly associated with increased informal and formal ACP [17]. NFs pointed out, that some patients were reluctant to accept the intervention and the attitude and communication habits regarding difficult topics like death and dying, remain challenging in ACP processes.

NFs observed that patients with lower socio-economic status had difficulties to fully understand and participate in the intervention. They described patients’ need for easy-to-read advance directive forms underlining the importance of documentation and indicating a limitation of the STADPLAN intervention. Limited health literacy has been identified as important barrier to ACP before and an intervention containing an easy-to-read advance directive in combination with an online ACP programme has resulted in significantly higher rates of ACP documentation [18].

The preparedness of caregivers in our study to participate in patients’ ACP ranged from decline to active engagement. A meta-synthesis exploring the role of caregivers in ACP found that caregivers want to get involved in ACP and decision-making according to patients’ wishes can be supported if healthcare professionals succeed in doing so. To be successful, caregivers individual understanding of ACP as well as communicational patterns and established relationships in the family need to be considered and patients must agree to caregivers’ involvement [6].

Nurses in our study identified their individual preferences for conversations and an empathetic personality as pivotal for choosing a role as ACP facilitator. As important motivation to participate they emphasised their expectation to develop personal skills which help them to meet patients’ needs regarding ACP. The compact programme in this study enabled nurses to raise awareness and initialise ACP. Still, they also mentioned insecurity and the need for comprehensive knowledge to act as facilitators. ACP facilitators need excellent communication skills and knowledge to successfully provide ACP [3]. Therefore, to guide a comprehensive ACP process including completion of documents, an extended education is required.

### Organisational level

Not surprisingly, a prominent aspect of organisational barriers was lack of time and staff, as has been reported previously [19]. Recruiting HCS for the STADPLAN study was challenging: many HCS declined participation due to lack of resources although they welcomed research in their setting. Still, most participating HCS managed the implementation of the intervention well and were able to provide qualified nursing staff. Considering the high workload and tight financial situation of home care services we designed a compact educational programme to ensure feasibility in this setting. Therefore, we aimed to build upon existing competencies and experiences of participating nurses which have been described as facilitators for ACP before [20].

By providing advanced roles for nurses, implementation of ACP might help to attract much needed qualified and motivated nurses to the home care setting.

### Macro level

The STADPLAN intervention focused on raising awareness, communication, and activating participants rather than on completing advance directives. NFs were aware of the excellent access their established relationship with patients provided, and how they could act as initiators and gatekeepers of ACP in the home care setting, a role which is associated with increased informal and formal ACP [17]. Yet, participants described ACP services and providers as fragmented at present. Services need to be better connected and easier to access to support HCS’ initiating role. Networking of ACP providers is already established in German statuary health insurance regulations (Hospice and Palliative Care Act [Hospiz- und Palliativgesetz]). For this, palliative care teams might have an important role as this was rated as the most successful current model for interprofessional collaboration by NFs and heads of HCS in the STADPLAN study.

### Strengths and limitations

This study has some strengths. We employed a variety of measures to ensure the quality of the research process, following key recommendations for the development and evaluation of complex interventions [11]. These recommendations focus on the *planning of the process evaluation*, the *design and conduct* of the study, the *analysis*, and the *reporting*. In the *planning of the process evaluation*, we defined the expertise and mode of collaboration between the study centres with main responsibility for process evaluation and intervention development respectively. All steps were discussed in the whole research group. Additionally, experts in the field (advisory board) were consulted to discuss research questions, design, and conduct in conjunction with the parent study.

For the *design and conduct* of the study, we developed a logic model that depicted the intervention, the participants, contextual factors, and mechanisms of change and selected main foci of the evaluation accordingly. Instruments and interview guides were developed based on this logic model and tested in a pilot study. Experienced researchers performed data collection and *analysis* and repeatedly reviewed and discussed results to ensure plausibility and consistency. As recommended by the UK MRC framework, we discussed the results of the process evaluation in advance to the main outcome measures [11]. To ensure high quality of *reporting*, we published a standalone study protocol as well as the results for the process evaluation adhering to specific reporting guidelines (SRQR [21], CReDECI 2 [22]). As proposed by the MRC framework, the results of the process evaluation are reported prior to the main results.

Our study has also some limitations. The process evaluation mirrored the overall recruitment difficulties in the parent trial. The main limitation is that we were not able to collect subsamples for quantitative process data randomly, thus we cannot estimate comparisons between groups and timepoints. An additional positive selection of participants in the process evaluation as compared to the main studies population can be assumed. Yet, comprehensive multi-perspective data enabled data-triangulation and validation of qualitative results, yielded detailed descriptions of change mechanisms, and illuminated a spectrum of contextual factors. The results refer to the specific aim and scope of the STADPLAN intervention focusing on raising awareness for ACP and initiating a process of reflection and communication. Interventions employing guided conversations can target four phases of an ACP process: preparation, initiation, exploration, and action [23]. The STADPLAN intervention primarily targets the first phase of preparedness. These characteristics need to be considered when comparing the reported results to other studies.

## Conclusions

The STADPLAN trial provides important evidence and implications for research and practice of nurse-led ACP interventions in care-depending community dwelling older people. Qualitative data indicate how contextual factors on all levels are crucial for the successful implementation of such an ACP intervention. To determine the exact effect of specific contextual aspects, these data must be assessed in the whole study population. As resources in research are limited and participants should not be overburdened by excessive data collection it remains a challenge to identify a core set of contextual factors relevant for a specific intervention and methods to explore them. The process evaluation presented here shows how a logic model can be employed to guide the entire research process.

Despite initial difficulties in recruiting home care services, we received positive feedback on the conduct and practical use of the study. Nurses and heads of HCS were highly motivated and provided an excellent access to an important target group for healthcare services. Further research in home care is therefore promising and might support the development of advanced roles for nurses with expanded competencies in this setting.

Implications for practice underline areas for further action. Firstly, a financial basis for ACP interventions in home care must be established as HCS currently have no spare resources for activities exceeding their core services. Unless this is remedied, a widespread implementation of the intervention seems not realistic. Secondly, nurses and other healthcare professionals need additional education to act as ACP facilitators as proficient communication skills and knowledge of medical and legal aspects are needed. For both aspects existing structures for ACP in residential care in Germany can be adapted. Thirdly, interprofessional collaboration in home care, especially with primary physicians and social services should be intensified. Access to ACP currently is, at least in Germany, fragmented. The responsibility of health care providers is to create joint services lowering the access threshold to ACP especially for care-dependent people.

## Supplementary Information

**Additional file 1.**

## Data Availability

Interview topic guides: Additional file 1. Interview data: Due to the sensitive nature of the questions asked in this study, participants were assured raw data would remain confidential and would not be shared. Workshop material, ACP conversations’ topic guides and information brochure: These data are available (in German) from the corresponding author (Mail to katharina.silies@uksh.de) on reasonable request.

## Abbreviations

ACP: Advance care planning
BEVA: German acronym for nurse facilitator (“Begleiter*in für vorausschauende Versorgungsplanung in der ambulanten Pflege”)
HCS: Home care service
NF: Nurse facilitator
STADPLAN: Study on advance care planning in care dependent community-dwelling older persons in Germany
