# Supplementary material for: Contextual factors influencing advance care planning in home care: process evaluation of the cluster-randomised controlled trial STADPLAN
Source: BMC Geriatr. 2022 Apr 20;22:345. doi: 10.1186/s12877-022-03026-2 (PMC9020149; doi:10.1186/s12877-022-03026-2)
Supplement: Supplementary file 1 — Additional file 1. [file 12877_2022_3026_MOESM1_ESM.pdf]

Contextual factors influencing advance care planning in home care: process evaluation of the cluster-randomised controlled trial STADPLAN

Additional file 1

Content

1. Topic guide for heads of home care services at baseline 2
2. Topic guide heads of home care services at t2 4
3. Topic guide nurse facilitators at baseline 6
4. Topic guide focus groups with nurse facilitators 8
5. Topic guide caregiver baseline 11
6. Topic guide caregivers t1 (post intervention) 13
7. Questions on self-efficacy regarding conversation on ACP (NF, post workshops day 1 and 2) 16
8. Questions on knowledge of ACP (NF, patients) 17
9. Code systems of qualitative data sets 18

9.1. Caregivers t0 18

9.2. Caregivers t1 18

9.3. NF focus groups 19

9.4. Heads of HCS t0 19

9.5. Heads of HCS t2 20

9.6. NF/staff t0 21

1. Topic guide for heads of home care services at baseline

Introduction

The STADPLAN study deals with the topic of advance care planning. By this we mean a repeated communication between patients, possibly their relatives and a specialist (e.g., family doctor, ...) about their wishes regarding medical treatment and care in situations in which they can no longer make decisions or speak for themselves. These wishes can be specified in advance directives or other documents.

At the beginning of our study, we would like to find out how things are generally on this topic. We have a few questions that relate to your care service and your customers (patients). The interview is recorded and then transcribed.

Information on data protection and consent to participate

The audio recording will be saved, and the transcript will be anonymised. The audio recording will be deleted immediately after the transcription and control has been completed. You can request viewing and deleting the data until the anonymous transcription has been completed. After that, it is no longer possible to connect the data to your person. The data is collected exclusively for the STADPLAN study. Only those involved in the study have access to the data. These persons are bound to secrecy. The data is protected from unauthorized access. The requirements of the data protection laws are fulfilled. The results of the survey will be published in the form of scientific papers. No conclusions can be drawn about individual persons.

Participation is voluntary. You can refrain from participating or revoke your consent at a later point in time without fear of negative consequences, but only until the data has been completely anonymised. Do you agree to the participation and the processing of your personal data?

Topic 1 – Advance Care Planning

To what extent is advance care planning an issue for you or your patients?

> What needs do the patients and their relatives have?
>
> What offers can you or your home care service make?
>
> In your opinion, which factors are beneficial or hindering in serving the needs of patients and their relatives?

How do you experience decision-making for patients who can no longer decide for themselves in the families?

> What do the patients and their relatives talk to you about?
>
> What role do the relatives play?
>
> What role does the family doctor or other caring specialists play?

Do you have experience with situations in which decisions had to be made on behalf of patients and in which written advance directives had to be used?

> If so, how did it go?
>
> Were the documents helpful in making the decision?

Were the documents specific enough about the situation to which they apply and about the measures that may be taken?

How do you experience the last phase of your patients' life in general?

What is difficult?

What do you experience as positive?

Topic 2: Conduct of the study

What made you decide to take part in the STADPLAN study?

What do you expect from the study for your facility or your home care service?

What do you expect for your patients and relatives?

How will you organize your staff's participation in the study?

How many employees can participate?

How was your staff's interest in participating in the study?

How do you handle the exemption or compensation for the additional work of your employees?

At this point, is there anything else you would like to add?

What other questions or suggestions would you like to speak about?

Thank you for the interview!

2. Topic guide heads of home care services at t2

Introduction

The STADPLAN study dealt with the topic of advance care planning.

At the end of our study, we would like to find out how things are now with this subject. We have a few questions that relate to your care service and your customers (patients). The conversation is recorded and then translated into a written text.

Information on data protection and consent to participate

The audio recording is saved and transcribed anonymously. The audio recording will be deleted immediately after the transcription and control has been completed. You can request viewing and deletion of the data until the anonymous transcription has been completed. After that, it is no longer possible to relate data to your person. The data is collected exclusively for the STADPLAN study. Only those involved in the study have access to the data. These persons are bound to secrecy. The data is protected from unauthorized access. The regulations of the data protection laws are observed. The results of the survey will be published in scientific papers. No conclusions can be drawn about individual persons.

Participation is voluntary. You can refrain from participating or revoke your consent at a later point in time without fear of negative consequences, but only until the data has been completely anonymised. Do you agree to the participation and the processing of your personal data?

Topic 1: Advance care planning

How do you experience the last phase of your patients' life in general?

> What is difficult?
>
> What do you experience as positive?

To what extent is advance care planning an issue for you or your patients?

> What needs do the patients and their relatives have?

How do you experience decision-making on behalf of patients who can no longer decide for themselves in the families?

> What do the patients and their relatives talk to you about?
>
> What role play
>
> … family members?
>
> ... the family doctor or other caring specialists?
>
> ... pension documents?
>
> How binding is it?

Do you have experience with situations in which decisions had to be made on behalf of patients and in which written advance directives had to be used?

> If so, how did it go?
>
> Were the documents helpful in making the decision?

Were the documents specific enough about the situation to which they apply and about the measures that may be taken?

To what extent has anything changed in the last few months since the start of the STADPLAN study?

... in terms of advance care planning?

... in relation to surrogate decision-making?

... in relation to your range of services?

What was positive?

Have you had any negative experiences?

Was something missing?

Topic 2: study context

Now I have a few more questions about your view of the STADPLAN study:

If you think back, what did you expect from the study for your facility or for your home care service?

To what extent have your expectations been met or not?

If not, what was missing?

What did you expect for your patients and their relatives by participating in the study? To what extent have your expectations been met or not?

If not, what was missing?

How were you able to organize your employees' participation in the study?

How many employees were able to participate?

How was your staff's interest in participating in the study?

How did you handle the exemption or compensation for the additional work of your employees?

All in all, how do you rate the study period overall in retrospect?

What was particularly worth mentioning?

What worked well

What not?

Do you have plans to offer your patients something for advance care planning even after the STADPLAN study has ended?

For example, consultations, information materials or referral to other providers?

At this point, is there anything else you'd like to add?

Thank you for the interview!

3. Topic guide nurse facilitators at baseline

Introduction

The STADPLAN study deals with the topic of advance care planning. By this we mean a repeated exchange between patients, possibly their relatives and a specialist (e.g., family doctor, ...) about their wishes regarding medical treatments and care in situations in which they can no longer decide for themselves. These wishes can be specified in advance directives or other documents.

With this interview we ask for your opinion on this topic. This information is important for us to get an idea of the general situation with the subject of advance care planning in outpatient care. The conversation is recorded and then written down.

Data protection information and consent to participate

The audio recording is saved and transcribed anonymously. The audio recording will be deleted immediately after the transcription and control has been completed. You can request viewing and deletion of the data until the anonymous transcription has been completed. After that, it is no longer possible to relate data to your person. The data is collected exclusively for the STADPLAN study. Only those involved in the study have access to the data. These persons are bound to secrecy. The data is protected from unauthorized access. The regulations of the data protection laws are observed. The results of the survey will be published in the form of scientific papers. No conclusions can be drawn about individual persons.

Participation is voluntary. You can refrain from participating or revoke your consent at a later point in time without fear of negative consequences, but only until the data has been completely anonymized. Do you agree to the participation and the processing of your personal data?

Topic 1: Advance Care Planning

How do you experience the last phase of your patients' life in general?

> What is difficult?
>
> What do you experience as positive?

To what extent is predictive care planning a professional topic for you or for your patients?

> What needs do the patients and their relatives have?
>
> What offers can you or your home care service make?
>
> How satisfied are you with it?

How do you experience decision-making for patients who can no longer decide for themselves in the families?

> What do the patients or their relatives talk to you about?
>
> What role do the relatives play?
>
> What role does the family doctor or other health care professionals play?

Do you have experience with situations in which decisions had to be made on behalf of patients and in which written advance directives were used?

> If so, how did it go?

Were the advance directives helpful in making the decision?

Were the advance directives specific enough in relation to the situation to which they apply and in relation to the measures that may be taken?

Topic 2: Personal attitude and role of nurses

How do you personally see the topic of advance care planning?

To what extent have you already dealt with it?

How do you rate your knowledge of this?

Do you already have professional experience with interviewing or advice?

If so, in what way?

What role can nurses play in advance care planning for their patients?

In what ways should or could nurses participate?

What could be helpful or even a hindrance when it comes to discussions (between nurses and patients or relatives) about advance care planning?

What effects do you expect from the planned ACP conversations in the study for the patients and their relatives?

What made you decide to take an active part in the STADPLAN project?

What are your hopes for the study as a whole?

What would you like to learn from the study?

At this point, is there anything else you would like to add?

Thank you for the interview!

4. Topic guide focus groups with nurse facilitators

Introduction

At the end of this 2nd workshop day, we ask you to give your opinion of the STADPLAN study as a whole and report on your personal experiences. Some questions may have been discussed earlier today. Nevertheless, your details are very important for us at this point to get a picture of how the study has gone from your point of view so far. To be able to evaluate this scientifically, we need documentation of your statements. That is why the group discussion is recorded and then written down so that we can evaluate it in a structured manner.

Information on data protection and consent to participate

The audio recording is saved and transcribed anonymously. The audio recording will be deleted immediately after the transcription and control has been completed. You can request viewing and deletion of the data until the anonymous transcription has been completed. After that, it is no longer possible to relate data to your person. The data is collected exclusively for the STADPLAN study. Only those involved in the study have access to the data. These persons are bound to secrecy. The data is protected from unauthorized access. The regulations of the data protection laws are observed. The results of the survey will be published in the form of scientific papers. No conclusions can be drawn about individual persons.

Participation is voluntary. You can refrain from participating or revoke your consent at a later point in time without fear of negative consequences, but only until the data has been completely anonymized. Do you agree to the participation and the processing of your personal data?

Topic 1: Workshops

At the beginning I would like to give you the opportunity to evaluate the workshops for nurse facilitators.

> If you think back to the first day of the workshop, what did the training day do for you personally?
>
> How do you rate the knowledge about predictive care planning that you learned from the training?
>
> How well did you feel prepared for the interviews?
>
> How well did you feel motivated to conduct the interviews?
>
> After you had the ACP conversations, was there anything that was missing from the first day of the training?

How did you feel about today's training day?

> To what extent were things picked up today that you may have missed after the first day?

Topic 2: Perception of the ACP conversations

Against the background of your role as nurse facilitator: how did you personally perceive the conversations with the patients?

> How well were you able to implement the discussions?

What was difficult, what went well?

How did you feel about your role in the conversation?

How is or was the STADPLAN project seen by your colleagues?

How do your colleagues think about the project and your role as BEVA?

How do you feel about your management's handling of the project? Do you feel supported?

Are / were there any difficulties?

How do you rate the experience of the patients and their relatives with the discussions?

Which topics were important to them?

How did the patients participate in the conversation, how the relatives?

How did you experience the interaction (collaboration / mutual influence) in the family?

How did you personally deal with it?

When you think about what you had hoped for your patients from the conversations - to what extent have your expectations been met or not?

If not, why?

What about the relatives, what changes have the conversations brought about for them?

All in all, how useful did you personally find these conversations?

Topic 3: Intervention components topic guide and manual

How did you use the topic guides in the interviews?

Did the planned course of the interview guides fit?

To what extent do you think the interview guides should be changed at any point?

How did you find the brochure "My Advance Care Planner"?

How did you incorporate the manual into the conversation?

What changes would you make to the brochure?

How did the patient use the "My Advance Care Planner" brochure?

How was the brochure used by the patients and their relatives?

In your opinion, how understandable and helpful was the brochure for patients and their relatives?

Topic 4: Conduct of the study and feasibility

How was the organization and implementation of the conversations overall?

What was difficult, what worked well?

Were relatives present at the second interview?

In what surroundings did the conversations take place? Was it possible to have an undisturbed conversation?

How do you rate the feasibility of such discussions in outpatient care overall?

Are the requirements feasible?

What difficulties did you encounter?

What went well

Is there anything else you would like to say at this point?

Thank you for the interview!

5. Topic guide caregiver baseline

Introduction

With this interview we would like to use key questions to discuss your experiences with and opinion of advance care planning. We also ask you for some personal information. The conversation is recorded and then translated into a written text.

Data protection information and consent

The audio recording is saved and transcribed anonymously. The audio recording will be deleted immediately after the transcription and control has been completed. You can request viewing and deletion of the data until the anonymous transcription has been completed. After that, it is no longer possible to connect data to your person. The data is collected exclusively for the STADPLAN study. Only those involved in the study have access to the data. These persons are bound to secrecy. The data is protected from unauthorized access. The regulations of the data protection laws are observed. The results of the survey will be published in the form of scientific papers. No conclusions can be drawn about individual persons.

Participation is voluntary. You can refrain from participating or revoke your consent at a later point in time without fear of negative consequences, but only until the data has been completely anonymized. Do you agree to the participation and the processing of your personal data?

Topic 1: Advance care planning

The STADPLAN study deals with the topic of advance care planning. By this we mean a repeated exchange between patients, possibly their relatives and a specialist (e.g., family doctor, ...) about their wishes regarding medical treatment and care in situations in which they can no longer decide for themselves. These wishes can be specified in advance directives or other documents.

At the beginning of our study, we would like to find out how things are in general with the subject of advance care planning. We have a few questions for you about this.

How do you feel about advance care planning, or have you already thought about your own wishes for treatments if you can no longer decide for yourself? For the "emergency case", so to speak?

> How sure are you that you could talk to others about your wishes today?
>
> How do you rate your willingness to make decisions about your own care planning?
>
> How do you currently rate your willingness to make decisions about your care planning in writing?
>
> Have you already found out about living wills, power of attorney and care will?
>
> Or: do you already have a Living will or power of attorney?
>
> If not: why?

Who would you like to talk to about how they would like to be treated in an emergency, when you can no longer express yourself?

> Or who did you talk to about it?

What role could nurses play in this?

What are the benefits of talking to a nurse about it?

What could speak against it?

When you think of the discussions on advance care planning that are carried out as part of the STADPLAN study, what are your expectations of them?

What do you expect or hope for your relatives in need of care?

Do you also have fears? If yes, which?

What do you expect or hope for yourself personally?

Do you also have fears? If yes, which?

What topics should be discussed?

Have you had an experience in which you were seriously ill and had to make difficult decisions about medical interventions?

Or have you witnessed such experiences with loved ones?

How did that go?

What made the decision difficult?

What helped you

Do you make decisions about medical measures alone, or do you think that should ultimately be decided by the doctor?

How do you deal with your dependent relative when decisions about medical or care treatments have to be made for him or her?

How do you come to a decision?

Who makes the decision?

Have you already discussed with your relative how a decision should be made in an emergency if he / she is no longer able to do so?

What did you discuss?

Did you consult with others, and if so, with whom?

How did you feel about that?

How do you feel how well you know about these wishes?

At this point, is there anything else you'd like to add?

Would you be prepared to conduct such a telephone interview with me again if you and your relative had the conversation with the home care service?

Thank you for the interview!

6. Topic guide caregivers t1 (post intervention)

Introduction

With this interview we would like to use key questions to discuss your experiences with and opinion of advance care planning. We also ask you for some personal information. The conversation is recorded and then translated into a written text.

Information on data protection and consent to participate

The audio recording is saved and written anonymously. The audio recording will be deleted immediately after the transcription and control has been completed. You can request viewing and deletion of the data until the anonymous transcription has been completed. After that, it is no longer possible to establish a personal reference. The data is collected exclusively for the STADPLAN study. Only those involved in the study have access to the data. These persons are bound to secrecy. The data is protected from unauthorized access. The regulations of the data protection laws are observed. The results of the survey will be published in the form of scientific papers. No conclusions can be drawn about individual persons.

Participation is voluntary. You can refrain from participating or revoke your consent at a later point in time without fear of negative consequences, but only until the data has been completely anonymized. Do you agree to the participation and the processing of your personal data?

Topic 1: Advance care planning

The STADPLAN study deals with the topic of advance care planning. Now that ACP conversations were held between nurses, patients and relatives as part of the study, we are interested in how these conversations were experienced.

We are questioning the participating patients, but today we would like to talk about how you personally experienced it.

When you think of the discussions on advance care planning that were held as part of the STADPLAN study, how did you experience these discussions?

> To what extent have your expectations of the conversations been met or not?
>
> What did the discussions provide for your relatives in need of care?
>
> What did the conversations provide for you?
>
> What did you find particularly good?
>
> Did you miss something, if so, what?
>
> How well do you feel informed now about advance care planning?

How did you find talking to a nurse about the subject?

How did you experience the nurse in the conversation?

> What was good?
>
> What was less good?

How did you find the brochure "My Advance Planner"?

> How understandable did you find that?

How helpful or useful is this manual for you or your dependents?

What did you like in particular?

What did you miss?

After talking to the nurse, did you talk to your relative again about the subject?

In what way?

What did you discuss?

Did you take action in any way?

Have you spoken to others about it?

Did you seek further advice?

How do you deal with your loved one when decisions about medical or care treatments have to be made for him or her?

How do you come to a decision?

Who makes the decision?

In your opinion, what changes have resulted from the discussions in the STADPLAN study?

In terms of clarity about what your loved one wants when she / he can no longer decide?

To what extent do you think he or she has clarified his / her wishes?

To what extent do you think you have become clear yourself about his / her wishes?

To what extent have the conversations in the STADPLAN study affected your role in the relationship with your care-dependent relative?

On the role of caring or supporting him or her?

On the role of surrogate decision maker?

What is your opinion now on the subject of advance care planning?

To what extent has anything changed in relation to your personal considerations because of the discussions as part of the study?

To what extent have you already thought about your own wishes for treatments in case you can no longer decide for yourself?

How sure are you that you could talk to others about your own wishes today?

How do you rate your willingness to make decisions about your own care planning?

Or have you already written something down?

Since our last interview, have you had an experience in which you had to make difficult decisions (about medical treatment or care) due to a serious illness?

Or have you witnessed such experiences with loved ones?

How did that go

What made the decision difficult?

What helped you

Do you make decisions about medical measures alone, or do you think that should ultimately be decided by the doctor?

[Quantitative questions on the situation as caring relatives and satisfaction with the home care service]

At this point, is there anything else you would like to add?

Thank you for the interview and all the best!

7. Questions on self-efficacy regarding conversation on ACP (NF, post workshops day 1 and 2)

How capable do you feel to …

- … start conversations with your patients on difficult topics (dying, limited medical care, prolonging life)?
- … inform your patients about topics such as advance directives and powers of attorney?
- … inform your patients about the content and requirements of advance directives?
- … have conversations with your patients about their attitudes and perceptions regarding their own death and dying?
- … cope with statements from your patients like "I would like to die"
- … explain to your patients which criteria an advance directive must meet in order to be effective?
- … be able to deal with difficult conversations with your patients and / or their relatives on the subject of advance directives?
- … empathically discuss with your patients their own wishes and ideas on the subject of life limitation or life extension?

Answer categories ranging from 1 = very capable to 6 = not at all capable

8. Questions on knowledge of ACP (NF, patients)

Please tick only one answer per question!

1.	With a power of attorney, I can …
a)	Make decisions about future medical treatments.
b)	designate one or more person(s) as representative for different legal transactions. The representative can speak for the person from this point in time.
c)	determine who should be appointed as a guardian by a court in a future guardianship procedure.
d)	settle all points mentioned under a)-c).
2.	With an advance directive, I can …
a)	Make decisions about future medical treatments.
b)	designate one or more person(s) as representative for different legal transactions. The representative can speak for the person from this point in time.
c)	determine who should be appointed as a guardian by a court in a possible guardianship procedure.
d)	settle all points mentioned under a)-c).
3.	In order for an advance directive to be effective, it must ...
a)	Be checked by a notary.
b)	be written in a state capable of giving consent and signed by hand.
c)	be signed by a doctor.
d)	meet all criteria mentioned under a)-c).
4.	In a situation in which an adult is unable to speak for himself (i.e. is unable to make decisions) …
a)	the next of kin (e.g. spouse or children) can make decisions about medical treatment for this person at any time.
b)	Only court guardians or authorized persons can make decisions on medical treatment for this person.
c)	The next of kin can only make decisions about medical treatment together with the physician in charge of the person.
d)	all situations mentioned under a)-c) are correct.

9. Code systems of qualitative data sets

9.1. Caregivers t0

Code-system Caregivers t0
1. Context caregivers
1.1.1. Decision-making
1.1.2. Knowledge and information on ACP
1.1.3. Attitudes towards ACP
1.1.4. Satisfaction with the HCS
2. Context dyad
2.1.1. Situation as caregiver
2.1.2. Relationship and communication in the dyad
2.2. Decision-making in the dyad
3. Intervention
3.1. Nurses as ACP facilitators
3.2. Expectations regarding the ACP conversations

9.2. Caregivers t1

Code-system t1
1. Macro context
2. Context patients
3. Context caregivers
3.1. Decision-making
3.2. Knowledge and information on ACP
3.3. Attitudes to and Experiences with ACP
3.4. Satisfaction with the HCS
4. Context dyad
4.1. Situation as caregiver
4.2. Relationship and communication in the dyad
4.3. Decision-making in the dyad
5. Intervention
5.1. How was the conversation for the patient?
5.2. How was the conversation for the caregiver?
5.3. Role of the nurse facilitator
5.4. Information brochure
5.5. Changes induced by the intervention

9.3. NF focus groups

Nurse facilitator (focus groups, t1)
1. Implementation
1.1. Assessment of the workshops
1.2. Topic guides for ACP conversations
2. Intervention
2.1. Impact on NF personal attitude towards ACP
2.2. Implementation and feasibility
2.3. Conversations
2.4. Information brochure
3. Participants (patients and caregiver)
4. Context meso level
5. Context macro level

9.4. Heads of HCS t0

Heads of HCS t0
1. Context STADPLAN study
2. Context heads of HCS/NF
2.1. Personal attitude towards ACP
2.2. Expectations and wishes regarding the study
3. Context patients
3.1. Physical, cognitive and emotional factors
3.2. Knowledge and assumptions regarding ACP
3.3. Attitudes of patients
3.4. Current status of patients‘ ACP
4. Context caregivers
5. Context dyad
6. Context meso level
6.1. End-of-life in homecare
6.2. Experiences with acute situations
6.3. Implementation and feasibility of ACP conversations
6.4. ACP in the HCS
7. Context macro level

9.5. Heads of HCS t2

Heads of HCS t2
1. Context STADPLAN study
2. Context head of HCS
3. Intervention
3.1. Emotional perception of the ACP conversations by patients
3.2. Observed effects of the intervention
3.3. Suggestions for adaptations
4. Context patients
4.1. Patients' needs regarding ACP
4.2. Physical, cognitive and emotional factors
4.3. Knowledge / assumptions
4.4. Patients' attitudes
4.5. Current status of patients' ACP
5. Context caregivers
6. Context dyad
7. Context meso level
7.1. End-of-life in homecare
7.2. Experiences with acute situations
7.3. Implementation and feasibility of ACP conversations
7.4. ACP in the HCS
8. Context macro level
8.1. Aspects of the macro level
8.2. Aspects of the pandemic

9.6. NF/staff t0

NF / staff t0
1. Context STADPLAN study
2. Context NF
2.1. Expectations and aspirations
2.2. Attitudes and knowledge of the future NF
2.3. Experiences in consulting
2.4. Experiences with acute situations
3. Context patients
3.1. Knowledge / assumptions
3.2. Physical, cognitive and emotional factors
3.3. Attitudes
3.4. Current status of patients' ACP
4. Context caregivers
5. Context meso level
5.1. End-of-life in home care
5.2. Current tasks and role of nurses in ACP
5.3. Feasibility of ACP as nurses' task
6. Context macro level
